# Bioactive Metabolite from Endophytic *Aspergillus versicolor* SB5 with Anti-Acetylcholinesterase, Anti-Inflammatory and Antioxidant Activities: In Vitro and In Silico Studies

**DOI:** 10.3390/microorganisms11041062

**Published:** 2023-04-19

**Authors:** Mohamed E. Elawady, Ahmed A. Hamed, Wamedh M. Alsallami, Ebtsam Z. Gabr, Mohamed O. Abdel-Monem, Mervat G. Hassan

**Affiliations:** 1 Microbial Biotechnology Department, National Research Centre, El-Buhouth St. 33, Cairo 12622, Egypt; mohamed_elawady82@yahoo.com; 2 Microbial Chemistry Department, National Research Centre, El-Buhouth St. 33, Cairo 12622, Egypt; 3 Botany and Microbiology Department, Faculty of Science, Benha University, Benha 13511, Egypt

**Keywords:** *Aspergillus versicolor*, bioactive metabolites, in silico study

## Abstract

Endophytic fungi are a highly unpredictable group of microorganisms that can create a diverse range of secondary metabolites with biological activity. These metabolites enhance the host’s ability to tolerate stress caused by various factors, such as disease, insects, pathogens, and herbivores. The secondary metabolites produced by endophytic fungi may have potential applications in agriculture, pharmacy, and medicine. The purpose of this study was to examine the anti-acetylcholinesterase activity of secondary metabolites extracted from endophytic fungi. *Aspergillus versicolor* SB5 was one of the many endophytic fungi isolated from *Juncus rigidus* and identified genetically with accession number ON872302. Our study utilized fermentation and microbial cultivation techniques to obtain secondary metabolites. During the course of our investigation, we isolated a compound called Physcion (C1) from the endophytic fungus *Aspergillus versicolor* SB5. We subsequently identified that C1 possesses inhibitory activity against COX-2 and LOX-1, with IC50 values of 43.10 and 17.54 µg/mL, respectively, making it an effective anti-inflammatory agent. Moreover, we found that C1 also exhibited potent anticholinesterase activity (86.9 ± 1.21%). In addition to these promising therapeutic properties, our experiments demonstrated that C1 possesses strong antioxidant capacity, as evidenced by its ability to scavenge DPPH, ABTS, O2 radicals, and NO and inhibit lipid peroxidation. To further investigate the molecular mechanisms underlying C1 pharmacological properties, we employed SwissADME web tools to predict the compound’s ADME-related physicochemical properties and used Molecular Operating Environment and PyMOL for molecular docking studies.

## 1. Introduction

Many studies have focused on investigating the relationship between pathogenicity in plants and their associated microorganisms. However, through various analyses and studies on the microbial diversity related to different plant species, it has been suggested that only a small proportion of the microorganisms that interact with plants are actually harmful [1]. The bulk of the bacteria that reside inside plants contribute significantly to the health and development of the plant, even if they may sometimes be neutral [2,3].

Endophytes are microorganisms, such as fungi, actinomycetes, or mycoplasma, that reside within plant tissues. Over 200 genera and 16 phyla of bacterial species have been identified as associated with endophytes, with the majority belonging to the Actinobacteria, Proteobacteria, and Firmicutes phyla [4].

Endophytic fungi have been found to produce compounds with antimicrobial, antitumor, and antioxidant activities [5]. Moreover, endophytic fungi can also increase the host plant’s tolerance to abiotic and biotic stress, including drought, salinity, disease, and herbivores. Due to their potential applications in various fields, the study of endophytic fungi has received increasing attention in recent years [6].

Endophytes have demonstrated the ability to produce a diverse array of bioactive metabolites with promising applications in the food, cosmetics, pharmaceutical, and agricultural industries. Furthermore, these metabolites hold potential as therapeutic agents for treating a wide range of diseases [7,8]. The characterized secondary metabolites included alkaloids, benzopyranones, chinones, flavonoids, phenolic acids, quinones, steroids, saponins, tannins, terpenoids, tetralones, and xanthones, as well as several other functional groups [7]. Alzheimer’s disease (AD), a prevalent form of dementia, is a progressive neurological ailment that primarily affects the elderly [9]. The medial temporal lobe and neocortical structures of the brain are heavily impacted by the accumulation of amyloid-beta peptide (Aβ) [10,11].

Mild cognitive impairment (MCI) is a stage between older adults and Alzheimer’s disease (AD) that is not significantly impaired in daily life. Although not all MCI patients develop AD and remain cognitively stable for years, the rate of advancement is estimated to be between 10% and 15% every year [12]. The 2015 World Alzheimer Report revealed that approximately 46.8 million individuals worldwide suffered from dementia, with an estimated global social cost of $818 billion USD. Alzheimer’s disease (AD) is the most prevalent type of dementia, accounting for 60–70% of all dementia cases. Degradation in areas rich in cholinergic neurons, such as the nucleus basalis of Meynert, frontal cortex, anterior cingulate cortex, and posterior cingulate cortex, has been linked to memory loss, irritability, and apathy in individuals with Alzheimer’s disease, according to pathological data. Acetylcholine (ACh), which is closely linked to memory function, including memory encoding, consolidation, and retrieval, has been strongly associated with this disease [13].

The main objective of this study was to investigate the diversity of endophytic fungi in *Juncus rigidus*, a common shrub species found in the Wadi-El Natron Valley in Egypt, with the aim of identifying new species or strains that produce valuable bioactive compounds. This study focused on assessing the diversity of endophytic fungi found in various healthy tissues of *Juncus rigidus* and their potential anti-activities. This research is the first report to our knowledge to evaluate the biodiversity, phylogeny, and anti-acetylcholinesterase activity of endophytic fungi present in *Juncus rigidus*.

## 2. Materials and Methods

### 2.1. Sampling, Isolation, and Purification of Endophyte

Plant specimens of *Juncus rigidus* were procured from various sites in the vicinity of Wadi-El Natron Valley Lake. The stems, roots, and leaves of the plants were gathered. In order to reach the inner tissue surface, small portions of the plant samples were cut into minute sections under aseptic conditions and washed three times using sterilized seawater. Subsequently, each sample was provided with 5 mL of sterilized seawater and subjected to a 30-min incubation period at 30 °C in a reciprocating water bath [14]. A serial ten-fold dilution was made with sterile sea water and platted (100 µL) on prepared potato dextrose agar (PDA) medium (potato infusion, 200 g; dextrose, 20 g; agar, 20.0 g; and 1000 mL of 50% sea water, pH 6.0).

### 2.2. Preparation of Fungal Extracts

Commercial rice (100 g) and 50% natural seawater (100 mL) were used to prepare a solid medium. Eight endophytic fungal isolates (SB1–SB8) were then added to the mixture, which was fermented at 30 °C for seven days. Following incubation, the culture media of each isolate was extracted using ethyl acetate, and the resulting solution was decanted and filtered. The organic extracts were then condensed in a vacuum prior to the activation of biological anti-acetylcholinesterase mechanisms.

### 2.3. Identification of Most Potent Fungal Isolate

#### 2.3.1. Phenotypic Analysis

The identification of the chosen fungal isolate was based on its cultural and morphological traits, including its pattern of colony formation, morphology of conidia, and pigmentation [15].

#### 2.3.2. Genetic Confirmation of Fungal Isolate

Molecular identification of the fungal isolate with potent anti-cholinesterase activity was conducted by extracting genomic DNA using the Qiagen DNeasy Mini Kit in accordance with the manufacturer’s guidelines. PCR amplification was carried out using a pair of primers, namely ITS1 (5′-TCCGTAGGTGAACCTGCGG-3′) and ITS4 (5′-TCCTCCGCTTATTGATATGC-3′), to amplify the ITS region. The PCR reaction mixture consisted of 1 µg of fungal genomic DNA, 1 µL each of 20 µM primers, a mixture of 10 mM dNTPs, 2 units of Taq DNA polymerase enzyme, and 10 µL of 5× reaction buffer. The PCR thermal profile comprised an initial denaturation step at 94 °C for 5 min, followed by 35 cycles of denaturation at 94 °C for 30 s, annealing at 55 °C for 30 s, extension at 72 °C for 90 s, and a final extension step at 72 °C for 5 min. The PCR product was purified using the JeneJET purification kit from Thermo Fisher Scientific and was subsequently sent to Macrogen, a sequencing service provider in South Korea. The 18S rRNA gene sequence was then aligned with the BLAST tool available on the NCBI database (GenBank C) in Bethesda, MD, USA. The 18S rRNA gene sequences of the bacteria were deposited into the nucleotide sequence databases of GenBank. Finally, a phylogenetic tree was constructed using the neighbor-joining method in MEGA10 software.

### 2.4. Purification and Structure Elucidation of the Bioactive Compound

The initial purification was carried out via flash column chromatography, where a 7 cm diameter column filled with normal phase silica was used, and 3.8 g of the crude extract was applied at a ratio of 20:1 adsorbent (silica gel) to solute (crude extract). A total of 110 fractions, each containing 5 mL, were collected and subjected to thin-layer chromatography (TLC) to determine the fractions that contained the desired compounds. The most potent fraction was subsequently purified using a Sephadex LH-20 column. Structural elucidation was conducted using LC-Mass spectrometry (MS), which measures the mass-to-charge ratio (*m*/*z*) of charged particles (ions). Finally, Nuclear Magnetic Resonance (NMR) was utilized for immediate structural elucidation of the pure compounds.

### 2.5. Acetylcholinesterase Inhibition Efficacy Assay

The inhibitory efficacy against acetylcholinesterase was studied using eight crude extracts. The method described by Ingkaninan et al. was modified to measure enzymatic activity [16]. A volume of 500 μL of DTNB (3 mM), 100 μL of AChI (15 mM), 275 μL of Tris–HCl buffer (50 mM, pH 8), and 100 μL of sample at 10, 20, 40, 60, 80, and 100 μg mL^−1^ were added to a 1 ml cuvette and used as a blank. An enzyme solution containing 0.28 UmL^−1^ was used in the reaction instead of 25 μL of buffer. The reaction was monitored for 5 min at 405 nm, and the data presented are the average of three replicates. Eserine hemi sulfate was employed as a positive control and was tested at different concentrations than the samples. The concentrations of eserine tested were 0.01, 0.02, 0.04, and 0.08 μg mL^−1^ [17].

### 2.6. Evaluation of Anti-Inflammatory Activity

#### 2.6.1. In Vitro Lipoxygenase (LOX) Inhibition Assay

To investigate the anti-inflammatory activity of the samples against the LOX enzyme from *Glycine max* (type I-B), they were compared to a reference drug (ibuprofen) with some minor modifications. This experiment was carried out in accordance with Granica et al. [18]. A mixture of 100 µL soybean LOX solution (1000 U/mL in borate buffer solution, pH 9) and 200 µL borate buffer was prepared in 96 well plates, and different concentrations of the samples were added to the mixture to obtain a final concentration range of 125–0.98 µg/mL. The mixture was incubated at 25 °C for 15 min, after which 100 µL of linoleic acid (substrate) was added to start the reaction. The absorbance increase at 234 nm was monitored to determine the inhibitory activity. The inhibitory percentages were calculated according to the following formula:

(1) Inhibitory activity (%) = (1 − As/Ac) × 100

Here, As represents the absorbance when the test substance is present, and Ac represents the absorbance of the control.

#### 2.6.2. In Vitro Cyclooxygenase (COX 2) Inhibition Assay

To investigate the anti-inflammatory activity by inhibiting the COX 2 enzyme, the samples were tested at different concentrations. COX (EC 1.14.99.1) activity was measured by monitoring the reaction between N, N, N, N-tetramethyl-p-phenylenediamine (TMPD) and arachidonic acid, resulting in its oxidation. This assay was performed according to Ouchemoukh et al. [19]. The inhibitory activity was assessed by measuring the rise in absorbance at 611 nm. The inhibitory percentages were determined using the following formula:

(2) Inhibitory activity (%) = (1 − As/Ac) × 100

The effectiveness of extracts and the reference compound (Celecoxib) in preventing the activity of the cox-2 iso-enzyme was evaluated by determining the concentration that causes a 50% decrease in enzyme activity (IC_50_). This was calculated using absorbance values in the presence of the test substance (As) and the control (Ac).

### 2.7. Evaluation of Antioxidant Activities of Compounds

#### 2.7.1. DPPH Radical Scavenging Activity

The free radical scavenging activity of the samples at concentrations of 10, 20, 30, 40, and 50 µg/mL was measured using 1,1-diphenyl-2-picryl-hydrazil (DPPH^•^) following the method described by Ibrahim et al. [20]. A series of concentrations of both the sample and the standard material, ascorbic acid, were prepared in methanol. The absorbance of the samples was then measured at 517 nm using a spectrophotometer. The DPPH radical scavenging activity was calculated using the following equation:

(3) $$DPPH\;•\;scavenging\;effect\;(\%)\;=\;[(A_{0}\;-\;A_{1})/A_{0}]\;\times \;100]$$

where A_0_ is the absorbance of the control, and A_1_ is the absorbance in the presence of the sample.

#### 2.7.2. ABTS Radical Cation Scavenging Activity

The ABTS radical cation scavenging activity of various concentrations of samples (10, 20, 30, 40, and 50 µg/mL) was evaluated and compared to Ascorbic Acid at equivalent concentrations using the procedure outlined by Miller and Rice-Evan [20]. To determine the ABTS radical cation scavenging activity, the absorbance at 734 nm was measured, and the calculation was performed using the subsequent equation:

(4) ABTS radical cation scavenging activity (%) = [1 − (A sample/A control)] × 100

#### 2.7.3. Lipid Peroxidation in Ammonium Thiocyanate Medium

The ability of the sample to inhibit Lipid Peroxidation was evaluated by following the method of Gulcin et al. [21] with some modifications and was compared with ascorbic acid. The level of peroxide was determined by measuring the absorbance at 500 nm in a spectrophotometer on a daily basis. The percentage reduction in lipid peroxidation was determined using the subsequent equation:

(5) $$Lipid\;Peroxidation\;Inhibition\;(\%)\;=\;[1\;-\;(A_{0})/A1)]\;\times \;100$$

where A_0_ is the absorbance of the control reaction, and A_1_ is the absorbance in the presence of the sample or standard compounds.

#### 2.7.4. Ferrous Ions (Fe^2+^) Chelating Capacity

The Fe^2+^ chelating activity of the sample was calculated according to the method of Dinis et al. [22] and was evaluated by comparing it with a standard compound (ascorbic acid at the same conditions). The absorbance of the reaction was measured at 562 nm using a spectrophotometer. The formula used to determine the percentage inhibition of ferrozine-Fe^2+^ complex formation is as follows:

(6) $$Inhibition\;(\%)\;=\;[(A_{0}\;-\;A_{1})/A_{0}]\;\times \;100$$

where *A*_0_ represents the absorbance of the control, while *A*_1_ represents the absorbance in the presence of both the sample and standards.

#### 2.7.5. Superoxide Anion Scavenging Activity

The superoxide anion (O^2−^) scavenging activity of the sample was determined using the method described by Dinis et al. [22], superoxide anion was generated by combining 3 mL of Tris-HCl buffer (16 mM, pH 8.0), 1 mL of NBT solution (50 µM), 1 mL of NADH solution (78 µM), and 1 mL of the sample or standard solution of varying concentrations. The reaction was initiated by adding 1 mL of PMS solution (10 µM). The reaction mixture was incubated at 25 °C for 5 min, and the absorbance was measured at 560 nm using a spectrophotometer. A control was prepared following the same procedure but without the sample. The superoxide anion scavenging was calculated using the subsequent formula:

(7) $$O^{2\;-\;}\;scavenging\;\%\;=\;[(A_{0}\;-\;A_{1})/A_{0}]\;\times \;100$$

where *A*_0_ is the absorbance of the control, and *A*_1_ is the absorbance of the sample or standard samples.

#### 2.7.6. Nitric Oxide Radical Scavenging Activity

The ability of the tested material to scavenge NO• radicals was evaluated using sodium nitroprusside (SNP) and compared to standard materials such as Ascorbic Acid. The generation of NO• was achieved by adding SNP to an aqueous solution at physiological pH, which produces nitrite ions that can be measured using the Greiss reagent [23]. The reaction mixture, consisting of SNP (10 mM) in phosphate-buffered saline pH 7.4, along with 2 mL of the sample and standard compounds at varying concentrations, was incubated at 25 °C for 150 min. Following incubation, 1 mL of the reaction mixture was extracted and combined with 1 mL of Greiss reagent. The absorbance of these solutions was then measured at 540 nm against the corresponding blank solution to determine the scavenging activity of NO• radicals.

### 2.8. In Silico Predictions of ADME-Related Physicochemical Properties and Toxicity Prediction

The ADME-related physicochemical properties of the obtained compound were predicted using SwissADME web tools [24]. The ProToxii webserver was used to estimate in silico toxicity for compounds, as reported by Banerjee et al. [23].

### 2.9. Molecular Docking Studies

The isolated compound was proven by molecular docking experiments to inhibit acetylcholinesterase (AChE). Molecular Operating Environment (MOE, V2015) and PyMOL (2.5.4) were used to conduct a molecular docking investigation [25]. The three-dimensional structure was downloaded from the Protein Data Bank (PDB) with the code 1EVE (AChE). The optimal positions were deeply seated inside the target protein’s (enzyme) active site, displaying all favorable and significant interactions.

## 3. Results

### 3.1. Isolation of Endophytes

**Figure 1:**
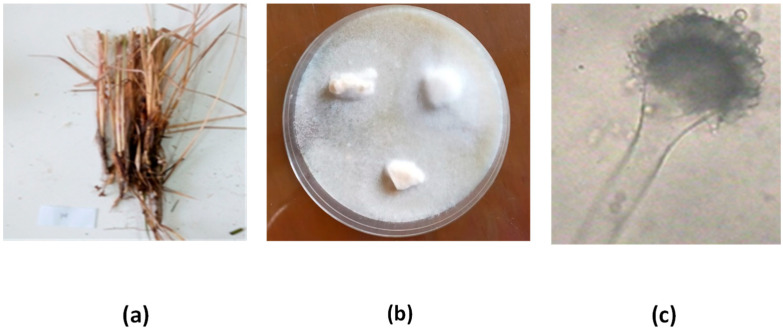
Isolation profile, (**a**) plant sample (*Juncus rigidus*), (**b**) isolation of fungi from *Juncus rigidus*, (**c**) Microscopic photo for morphological shape of *Aspergillus*.

Eight endophytic fungi (SB1–SB8) were obtained from a plant sample (*Juncus rigidus*) collected from Wadi El-Natron in El-Beheira Governorate, Egypt. The fungi were isolated from various parts of the plant, including leaves, stems, and roots, on PDA medium (Figure 1).

### 3.2. Identification of the Most Potent Endophytes

Based on the biological screening of all isolated endophytes for anticholinesterase activity (as shown in Table 1), the most potent isolate (SB5) demonstrated anticholinesterase activity of 79.5 ± 1.39%. Therefore, it was selected and identified morphologically and genetically using 18s rRNA gene techniques. The colonies reached 5–6 cm diameter in 7 days at 28 °C on Czapek medium, exhibiting pale greenish-yellow colonies with deep green colonies at the margins, and a yellow to brown reverse side with age. The conidial head was found to be radiate, with a conidiophore diameter of 15.0 μm. The globose-subglobose vesicle measured 29.0 μm, while the sterigmata were uniseriate or biseriate, with primary conidia of 7.8 × 4.5 μm. The conidia were observed to be globose and measured 5.5 µm.

**Figure 2:**
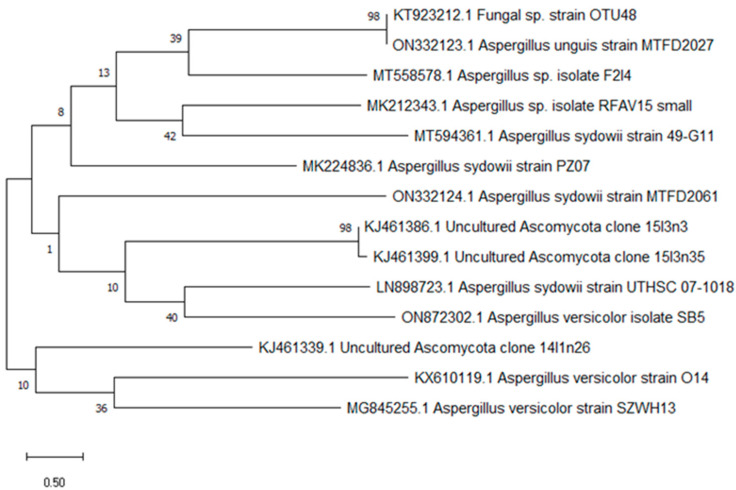
Constructed phylogenetic tree of *Aspergillus versicolor* SB5.

To determine the similarity score and statistical significance of matches, the 18S rRNA gene sequences of the isolate SB5 was retrieved, recognized, and compared to other identified sequences in the GeneBank database using the BLAST tool (https://blast.ncbi.nlm.nih.gov/Blast.cgi, accessed on 1 January 2023). The results revealed that the 18S rRNA gene sequence of SB5 and *Aspergillus versicolor* were very similar, showing 100% homology. Using the neighbor-joining technique, MEGA 10 was employed to create a phylogenetic tree and perform the analysis. After examining its DNA sequence and morphological characteristics, the SB5 strain was identified and classified as an alternative *Aspergillus versicolor* SB5. It was then deposited in GenBank and assigned the accession number ON872302.1, as shown in Figure 2.

### 3.3. Fermentation, Screening, and Structure Elucidation

*Aspergillus versicolor* SB5 was cultured on solid rice medium, and the resulting crude extract was purified through various chromatographic methods to isolate the specific secondary metabolite responsible for strong anticholinesterase activity, as shown in Table 2 and Table 3. Fractionation of the crude extract using flash column chromatography resulted in ten fractions, with fraction 3 exhibiting the strongest anticholinesterase activity (82.1 ± 0.75%). This fraction was further purified using Sephadex LH20, producing seven subfractions, the most potent of which was subfraction 5, exhibiting 86.9 ± 1.21% anticholinesterase activity. Structures of the compound (subfraction 5) were confirmed on the basis of different spectroscopic means (NMR, and MS) (see supporting information) and comparison with the corresponding literature. The compound was obtained as a deep orange fine crystal. ^1^H NMR spectra showed characteristic signals for aromatic protons at δ_H_ 6.71 (1H, s, H-7), 6.97 (1H, s, H-2), 7.62 (1H, bd, H-5), and 7.93 ppm (1H, bd, H-4). Two characteristic singles appeared in the aliphatic and oxygenated aliphatic regions at δ_H_ 2.58 ppm (3H, s, -CH_3_) and 3.87 ppm (3H, s, -OCH_3_). On the basis of its LC-MS/MS (positive ion mode) *m*/*z* 285.21[M+H]+ (calc for C_16_H_12_O_5_, 284.26), the compound was identified as Physcion (1,8-dihydroxy-3-methoxy-6-methylanthracene-9,10-dione) ***C1*** based on its chromatographic properties, proton, and available reported data [26].

### 3.4. Biological Evaluation

#### 3.4.1. Anti-Inflammatory Property of Isolated Compounds

The anti-inflammatory properties of C1, which was obtained from the *Aspergillus versicolor* SB5 fungus, were determined by assessing its inhibitory effects on COX-2 and LOX-1, using celecoxib and ibuprofen as reference drugs. However, the data presented in Table 4 show that C1 selectively inhibited COX-2. The inhibition of COX-2 was dependent on the concentration of C1, ranging from 26.42 ± 1.33% at 5 µg/mL to 74.80 ± 1.40% at 200 µg/mL. The results indicated that the IC_50_ value for COX-2 inhibition by C1 was 43.10 µg/mL, which was higher than that of celecoxib (IC50: 19.63 ng/mL). Moreover, C1 inhibited LOX-1, with inhibition ranging from 25.77 ± 1.22% at 5 µg/mL to 91.06 ± 1.74% at 200 µg/mL. The IC_50_ value for LOX-1 inhibition by C1 was 17.54 µg/mL, which was higher than that of the reference drug (IC_50_: 8.77 µg/mL).

#### 3.4.2. DPPH Free Radical Scavenging Ability

The scavenging potential of C1 for free radicals was evaluated using the DPPH assay and compared to that of ascorbic acid, as indicated in Table 5. The results demonstrated that C1 displayed promising free radical scavenging activity, which significantly increased with the rise in C1 concentration from 10 to 50 µg/mL (31.55 ± 1.82% and 83.61 ± 1.72%, respectively). In contrast, ascorbic acid showed 62.47 ± 1.02% scavenging activity, which rose to 99.13 ± 0.45% at the same two concentrations (*p* < 0.05). Based on the IC50 values, the scavenging activity of ascorbic acid was greater than that of *C1*, with significant differences observed (*p* < 0.05).

#### 3.4.3. ABTS Cation Radical Scavenging Capability

The scavenging ability of C1 and a reference drug against ABTS radicals was evaluated using the ABTS discoloration method at various concentrations, as shown in Table 6. C1 demonstrated activity at a low concentration of 10 µg/mL, exhibiting 40.41 ± 1.53% scavenging percentage, which gradually increased to 85.30 ± 0.93% as the concentration was raised to 50 µg/mL. In contrast, vitamin C showed 68.96 ± 1.25% and 99.16 ± 0.39% scavenging percentage for the same concentrations, respectively (*p* < 0.05). The IC50 of C1 in the ABTS system was 17.80 μg/mL, while ascorbic acid had an IC50 of 8.65 μg/mL.

#### 3.4.4. Reduction Capability

The ability of C1 to reduce Fe^3+^ was evaluated by the Fe^3+^–Fe^2+^ transformation test, and ascorbic acid was used as a reference material. As shown in Table 7, C1 had a moderate effect on Fe^3+^ reduction, which increased significantly with increasing concentrations. C1 showed a reductive ability with an absorbance value of 0.618 ± 0.04, which was lower than that of vitamin C (0.763 ± 0.02).

#### 3.4.5. Fe^2+^ Ion Chelation Ability

Table 8 presents the data obtained from the evaluation of the chelation efficacy of C1 and vitamin C on ferrous ions (Fe+) among the transition metals by generating complexes with ferrozine. The results showed that C1 had a moderate ability to chelate ferrous ions compared to ascorbic acid. At the lowest concentration, C1 had a chelation percentage of 40.17 ± 1.24, which increased to 67.91 ± 1.82% at the highest concentration, while vitamin C had 63.58 ± 1.05% and 98.08 ± 0.82%, respectively. The values of IC50 for C1 and vitamin C were 27.97 µg/ mL and 6.32 µg/ mL, respectively.

#### 3.4.6. Lipid Peroxidation Inhibition Capacity

Table 9 presents the results of the preventive effect of C1 on linoleic acid peroxidation. It was found that C1 inhibited the peroxidation of linoleic acid in a concentration-dependent manner, with the lowest inhibition activity of 47.89 ± 0.87% observed at the lowest concentration of 10 µg/mL, and the highest inhibition activity of 78.10 ± 1.52% observed at the highest concentration of 50 µg/mL. Ascorbic acid showed inhibition activities of 55.63 ± 1.20% and 95.78 ± 1.08% for the two concentrations, respectively. The concentration of C1 required to prevent 50% linoleic acid oxidation into peroxide was 15.58 µg/mL, while that of ascorbic acid was 8.42 µg/mL.

#### 3.4.7. O_2_ Radicals Scavenging Capacity

Table 10 displays the inhibition of generated SOR by C1 at varying concentrations and the comparison of the results to the reference material. C1 demonstrated a scavenging percentage of SOR ranging from 59.91 ± 0.99% at 10 µg/mL to 89.27 ± 1.51% at 50 µg/mL, while ascorbic acid displayed scavenging percentages of 68.34 ± 1.25% and 96.35 ± 1.97% at the same concentrations. In terms of the IC50 value, Physcion exhibited a lower value (7.44 µg/mL) than ascorbic acid (6.19 µg/mL).

#### 3.4.8. NO Scavenging Capacity

Using a SNP that creates a NO system, the capacity of C1 to scavenge NO radicals was measured. Based on the information presented in Table 11, it was found that *C1* had a weaker NO scavenging ability than the reference substance. However, this ability was dependent on concentration, and as the concentration of C1 increased over time, its ability to scavenge NO increased significantly. Specifically, at a concentration of 10 µg/mL, C1 was able to scavenge 37.99 ± 1.93% of the NO, while at a maximum concentration of 50 µg/mL, it was able to scavenge 70.55 ± 1.71% of the NO. Both of these values were lower than the corresponding values for ascorbic acid, which was able to scavenge 59.99 ± 1.12% and 95.78 ± 1.95% of the NO at the same concentrations, respectively. The amount of C1 required to capture 50% of the generated NO was 27.37 µg/mL, while for ascorbic acid, it was 8.01 µg/mL.

### 3.5. In Silico Predictions of ADME-Related Physicochemical Properties and Toxicity Prediction

**Figure 3:**
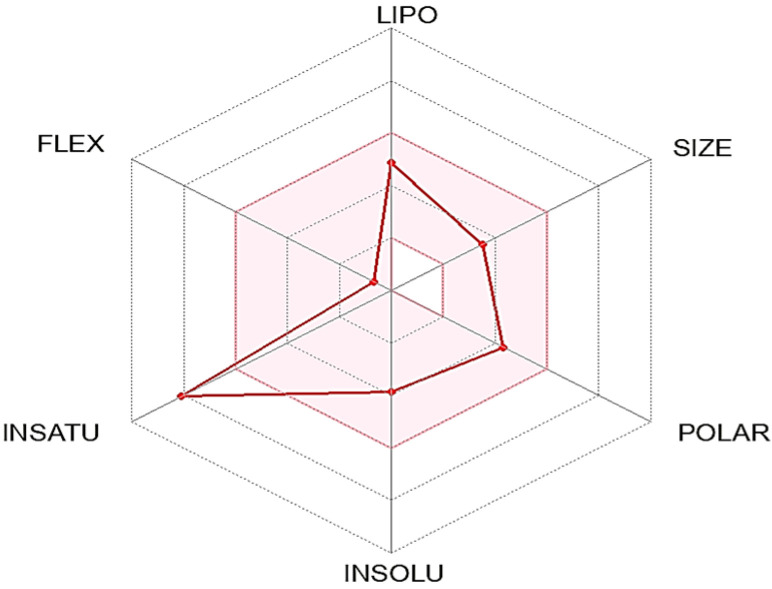
The bioavailability pink area indicates the optimal range for each property (lipophilicity: XLOGP3 between 0.7 and +5.0, size: MW between 150 and 500 g/mol, polarity: TPSA between 20 and 1302, solubility: log S not higher than 6, saturation: fraction of carbons in the sp3 hybridization not less than 0.25, and flexibility: no more than 9 rotatable bonds).

**Figure 4:**
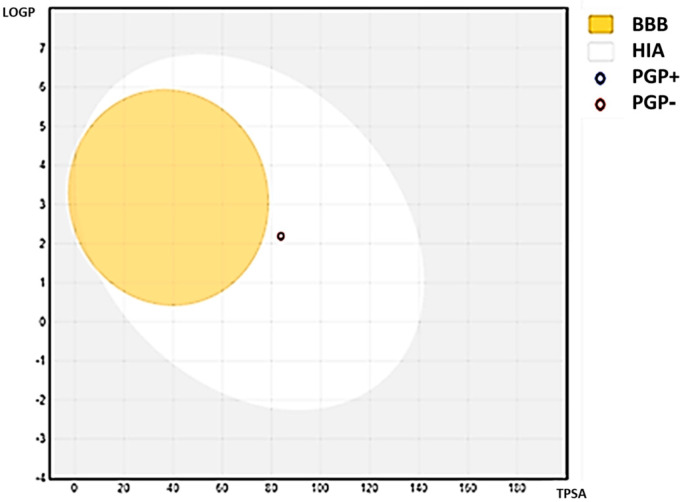
Physcion’s BOILED-Egg plot. The yellow zone (yolk) is for very possible BBB permeability, whereas the white region (GI) is for highly probable HIA (GI) absorption. Molecules with limited absorption and no brain penetration are shown by the outside gray area. The points are also colored blue if the P-gp substrate (PGP+) is anticipated and red if the P-GP non-substrate (PGP) is projected.

The ADME-related physicochemical properties of *C1* were determined via the SwissADME webserver [24]. The measurements are based on the created molecule’s characterization using drug-likeness criteria. As a result, the compound passed with flying colors through the Lipinski, Veber, and Ghose rules filter. It might be utilized as an oral medication since it has a 0.55 percent bioavailability in the mouth (Table 12). Additionally, the Bioavailability Radar map, which is based on six physicochemical properties, was used to estimate drug-likeness quickly. This plot is based on size, polarity, lipophilicity, solubility, flexibility, and saturation (Figure 3). The compound showed an ideal range (pink region) for all parameters except one, flexibility, according to the derived diagram. Lipophilicity, which indicates how permeable a drug is to molecules across cell membranes, is another crucial physicochemical metric [27,28]. The examined compound had Log Po/w values under 5, which was 2.27), which suggested excellent absorption and permeability across the cell membrane. Furthermore, solubility is one of the most important elements affecting a compound’s absorption throughout any formulation process [24]. The chemical is soluble, as per the ESOL topological model. The chemical satisfies the rule of three for medicinal chemistry and Leadlikness (RO3). The chemical had modest synthetic accessibility, with a value for the synthetic accessibility score (SAscore), which is based on fragment similarity and complexity penalties (2.69). Table 13 shows the compound pharmacokinetic parameters analyzed via the vector machine algorithm model [24]. The isoenzymes CYP1A2, CYP2C9, and CYP3A4 are specifically inhibited by the substance. Figure 4 derived from refs. [24,29] shows the BOILED-Egg model (Brain or Intestinal Estimate D permeation technique, WLOGP versus TPSA). In humans, this compound has a high gastrointestinal absorption rate (GI). The substances are non-PGP substrates (PGP-, red dots), and the blood–brain barrier (BBB) is permeable to them (TPSA 83.83), suggesting that they have an impact on the central nervous system (CNS) [28]. The generated compound has a log (Kp) of −5.88 cm/s; the higher negative the log Kp, the less skin permeant the compound is, according to Daina and Zoete [29], who discussed how to predict the skin permeability coefficient (Kp) of the produced compound.

On the other hand, the ProTox ii webserver was used to forecast the acquired compound’s toxicity [23]. The results in Table 14 demonstrate that the compound has no pronounced toxicity. had a low level of toxicity. The findings are consistent with research that found Physcion to be non-toxic to humans acutely [30].

### 3.6. Molecular Docking Study

**Figure 5:**
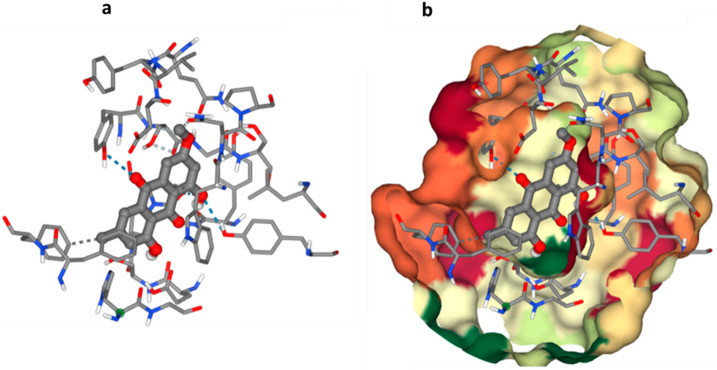
Possible binding modes of the obtained compound with Acetylcholinesterase (AChE); (**a**) Interaction of C1 with amino acid residues and (**b**) catalytic pocket and compound.

The Molecular Operating Environment (MOE) [23] was used to conduct generic molecular docking research in positive mode for the found bioactive molecule. Acetylcholine esterase’s three-dimensional structure may be found in the Protein Data Bank (PDB) under the code 1EVE (AChE). The PubChem database was used to obtain the chemical structure of the obtained molecule as smiles. As previously said, each one was presented separately to the MOE window to prepare for docking. Computational study showed the possible interaction of Physcion and Acetylcholinesterase (AChE) Figure 5 shows a conventional hydrogen bonding interaction for the O4 ligand atom with GLY 117, the O3 atom with TYR 121, and the O4 atom with TYR 30. Another type of interaction was also observed through hydrophobic contact of the C4 ligand atom with THR 10 and the C15 ligand atom with PHE 330, which may lead to disruption of the Acetylcholinesterase (AChE) function.

## 4. Discussion

Endophytic fungi are symbiotic microorganisms that live inside plants without causing any harm to the host plant. Endophytes may actively or passively stimulate plant development via a range of processes, resulting in higher host fitness and plant resilience to biotic and abiotic stressors. These fungi can produce a wide range of biologically active secondary metabolites that have various pharmacological properties. As a result, endophytic fungi have become an attractive source of novel bioactive compounds for drug discovery [31]. By inducing the solubilization of phosphorus, potassium, and zinc and inducing the host plant’s defensive response against phytopathogens via a variety of methods, fungal endophytes either directly or indirectly support the development of plants. Auxin, abscisins, ethylene, and gibberellins are a few examples of plant hormones that may be changed by these mechanisms, along with competition, niche exclusion, and siderophore synthesis [32]. Pathogens can also be directly antagonistic via antibiosis, parasitosis, or predation. The photochemistry of the host may be related to variations in endophytes throughout plants and tissues, according to prior research [33]. Based on the current findings, *Juncus rigidus* is a wild plant that harbors a significant number of endophytic fungi, which may be attributable to its greater active chemical concentration [34].

The goal of this research was to find a natural compound that could be used to treat Alzheimer’s disease. As a result of their unique living conditions, endophytic marine microorganisms frequently create bioactive compounds with novel activities and structures. Thus, a total of eight endophytic fungi were recovered from *Juncus rigidus*. Due to their tremendous structural variety and complexity, endophyte-isolated bioactive natural chemicals have played a significant role in the search for new therapeutics [35].

Isolation of endophytic fungi from plants is a crucial step in exploring the diversity of endophytic fungi and their potential as sources of novel bioactive compounds. The process of isolation involves surface sterilization of the plant samples to eliminate any epiphytic fungi or bacteria that may contaminate the samples. After surface sterilization, different plant parts, such as leaves, stems, and roots, are aseptically removed and cultured on suitable media. Several studies have reported the isolation of endophytic fungi from various plant species. For example, one study isolated endophytic fungi from the leaves and stems of the medicinal plant *Carpesium abrotanoides*. The study identified 41 endophytic fungal strains belonging to 18 different genera, and many of these strains exhibited antimicrobial and antioxidant activities [36]. Another study isolated endophytic fungi from the bark of the medicinal plant *Maytenus ilicifolia*. The study identified 102 fungal strains belonging to 28 different genera, and many of these strains exhibited cytotoxic and antifungal activities [37].

Furthermore, a study isolated endophytic fungus from the roots of the medicinal plant *Panax notoginseng*. The study identified 116 fungal strains belonging to 22 different genera, and many of these strains exhibited antioxidant and anti-inflammatory activities [37] Overall, the isolation of endophytic fungi from plants has the potential to lead to the discovery of novel bioactive compounds with various pharmacological properties. The diversity of endophytic fungi found in different plant species highlights the importance of exploring different plant sources for the isolation of these microorganisms.

The ability of ethyl acetate extracts from an isolated fungus to produce AChEIs was evaluated. Multiple isolates showed anticholinesterase activity, but only one endophytic fungal strain (SB5) was found to be the most effective, suppressing AChE at a rate of 79.5 ± 1.39. Fungal endophytes produce more secondary metabolites than other types of endophytic microbes. To identify the most potent endophyte, isolate SB5, the strain SB5 was identified visually and genetically using the 18s rRNA gene approach and was identified as Aspergillus versicolor SB5. It has been deposited in GenBank with accession number ON872302.1.

Fractionation and separation of the crude extract of *Aspergillus versicolor* SB5 was done by flash column chromatography and sephadex LH20 according to the AChE activity of the obtained fractions (Table 2 and Table 3). Then, the most potent compound was identified as Physcion (1,8-dihydroxy-3-methoxy-6-methylanthracene-9,10-dione) based on its chromatographic properties, mass analysis, and available reported data (Figure 3). Endophytic fungi have been shown to be a perennial and abundant source of antioxidant, antimicrobial, anticancer, and anticholinesterase compounds [5]. C1 constitutes an important class of natural compounds with a broad scope of pharmacological properties, including anti-bacterial, antioxidant, laxative, anti-tumor, and other activities. They may have also held a variety of pharmacological properties, including laxative, anti-tumor, anti-inflammatory, antibacterial, antioxidant, anti-injury, and acetylcholinesterase inhibitory properties, according to recent medical studies [38].

C1 obtained from the fungus *Aspergillus versicolor* SB5 was assayed for its inhibitory action against COX-2 and LOX-1. C1 inhibited COX-2 and LOX-1 with 74.80 ± 1.40% and 91.06 ± 1.74% at 200 µg/mL. Physcion being the main component of *Reynoutria elliptica* was likely to reduce the synthesis of NO and PGE2 via attenuating iNOS and COX-2 expression and inhibit TNF-α secretion by suppressing mitogen-activated protein kinases (MAPKs) and NF-κB activation [39]. By blocking the MEK/ERK pathway, Physcion was able to decrease Jurkat E6.1 cells’ CXCR4-mediated chemotaxis. It also reduced HSC-T6 cells’ chemotactic migration [40].

Additionally, endophytic fungi have recently been recognized for their ability to produce antioxidant compounds that have an anti-aging effect, protect hepatic cells from damage, improve the body’s defense mechanism, offer protection against digestive disorders, and reduce obesity. Antioxidants are a family of compounds considered to be the most effective against a variety of age-related conditions, such as Alzheimer’s disease. C1 capacity to scavenge free radicals was tested using different assays at different concentration (10–50 µg/mL) in comparison to Ascorbic acid as a reference drug at the same concentrations. The main pigments of *Stemphylium lycopersici*, Physcion, may be able to scavenge hydroxyl and ABTS radicals [41]. In contrast, it has been found by several studies that C1 exhibits clear activity in the ABTS and DPPH radical-scavenging assays [42]. It is interesting to note that C1 demonstrates anti-tumor action via causing ROS production in tumor cells, which is the reverse of its impact on free radicals. Furthermore, according to certain research, C1 produced pro-oxidant effects on human neutrophil cells and possessed DPPH and superoxide radical scavenging properties [43].

ADME properties are crucial in drug development, as they determine the drug’s effectiveness and safety in the body. Absorption refers to how a drug enters the bloodstream, and drugs can be formulated as prodrugs to optimize absorption. Distribution determines how the drug is transported to its target site and can be enhanced through targeted drug delivery systems. Metabolism involves how a drug is broken down by enzymes in the body and can be affected by genetic variations and drug interactions. Excretion refers to how the drug is eliminated from the body, primarily through the kidneys or liver, and can be influenced by the drug’s molecular weight, polarity, and ionization. By optimizing these ADME properties, drug developers can increase their chances of success in drug development and provide safe and effective treatments to patients [44]. In this study, the ADME properties of C1 were examined and analyzed.

Molecular docking studies have become increasingly important in drug discovery and development, allowing for the evaluation of ligand–receptor interactions at a molecular level. In the case of cholinesterase enzymes, the goal is to identify compounds that can act as inhibitors of these enzymes. This information can then be used to identify potential inhibitors for further study and optimization. However, it is important to note that molecular docking results should be validated through experimental methods, such as in vitro and in vivo assays, to confirm the compound’s effectiveness and safety. Overall, molecular docking studies provide a valuable tool in the early stages of drug discovery, aiding in the identification of potential drug candidates for further development [45].

## 5. Conclusions

The endophytic fungus *Aspergillus versicolor* SB5 was isolated from the plant *Juncus rigidus* growing in Wadi El-Natron at El-Beheira Governorate, Egypt. Its large-scale fermentation and working up of the crude extract, according to the anticholinesterase activity and based on its chromatographic properties, proton and carbon spectra, and available reported data, led to the isolation of Physcion. It displayed anti-inflammatory, antioxidant, and acetylcholinesterase inhibition activities. These findings highlight the importance of endophytic fungi as a source of bioactive secondary metabolites. 

## Figures and Tables

**Table 1:** Acetylcholinesterase inhibition (%) of different fungal extracts.

Extract	Acetylcholinesterase Inhibition (%)			
	50 µg	100 µg	200 µg	300 µg
SB 1	5.4 ± 1.72	13.6 ± 0.76	19.5 ± 1.04	25.9 ± 1.26
SB 2	0.0	0.0	0.0	0.0
SB 3	6.8 ± 1.43	12.5 ± 1.12	21.6 ± 1.34	29.3 ± 1.25
SB 4	24.7 ± 0.46	32.8 ± 1.54	41.4 ± 1.25	52.7 ± 0.54
SB 5	32.8 ± 1.22	38.0 ± 0.98	56.2 ± 1.21	79.5 ± 1.39
SB 6	21.6 ± 1.02	29.4 ± 0.79	38.8 ± 1.32	51.2 ± 1.28
SB 7	0.0	0.0	0.0	0.0
SB 8	7.3 ± 1.21	16.9 ± 1.11	25.4 ± 1.46	32.7 ± 1.17

**Table 2:** Acetylcholinesterase inhibition (%) of different fractions separated from SB5.

Extract	Acetylcholinesterase Inhibition (%)			
	50 µg	100 µg	200 µg	300 µg
Fraction 1	21.4 ± 1.13	28.7 ± 0.75	34.2 ± 0.98	42.8 ± 1.44
Fraction 2	0.0	0.0	0.0	0.0
Fraction 3	39.1 ± 1.66	51.2 ± 0.89	67.6 ± 1.59	82.1 ± 0.75
Fraction 4	25.9 ± 1.29	33.4 ± 1.37	46.8 ± 1.53	51.2 ± 1.36
Fraction 5	0.0	0.0	0.0	0.0
Fraction 6	0.0	0.0	0.0	0.0
Fraction 7	7.0 ± 1.16	13.8 ± 1.21	19.9 ± 1.40	25.4 ± 1.36
Fraction 8	0.0	0.0	0.0	0.0
Fraction 9	28.6 ± 1.64	42.1 ± 0.66	52.7 ± 1.43	63.6 ± 1.19
Fraction 10	0.0	0.0	0.0	0.0

**Table 3:**
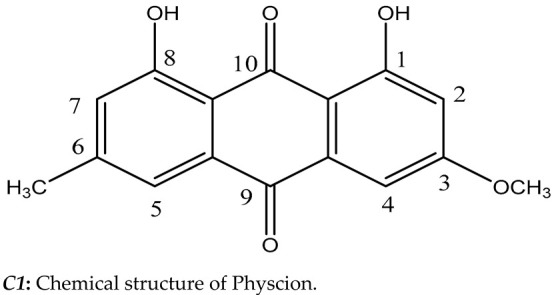
Acetylcholinesterase inhibition (%) of different sub-fraction from Fraction 3.

Extract	Acetylcholinesterase Inhibition (%)			
	50 µg	100 µg	200 µg	300 µg
Sub fraction 1	0.0	0.0	0.0	0.0
Sub fraction 2	0.0	0.0	0.0	0.0
Sub fraction 3	14.6 ± 1.34	27.3 ± 1.18	36.4 ± 0.73	41.8 ± 1.05
Sub fraction 4	7.9 ± 1.17	14.6 ± 1.15	26.2 ± 0.88	38.1 ± 1.36
Sub fraction 5	42.5 ± 1.41	55.6 ± 0.92	71.3 ± 1.25	86.9 ± 1.21
Sub fraction 6	0.0	0.0	0.0	0.0
Sub fraction 7	22.5 ± 1.06	34.7 ± 0.81	44.9 ± 1.61	51.2 ± 1.52

**Table 4:** COX-2 and LOX-1 inhibition by different concentrations of C1 and the standard drug.

LOX-1			COX-2		
Conc. (µg/mL)	Inhibition (%)		Conc. (µg/mL)	Inhibition (%)	
	C1	Ibuprofen		C1	Celecoxib
5	25.77 ± 1.22	42.16 ± 0.63	5	26.42 ± 1.33	32.95 ± 0.64
10	34.30 ± 1.52	57.32 ± 1.90	10	31.71 ± 1.72	38.47 ± 1.40
20	56.62 ± 1.46	70.12 ± 2.1	20	37.39 ± 0.79	51.35 ± 0.92
50	63.40 ± 0.82	78.34 ± 0.92	50	58.04 ± 1.12	66.44 ± 2.1
100	86.29 ± 0.89	89.21 ± 1.2	100	67.91 ± 1.64	72.73 ± 1.96
200	91.06 ± 1.74	95.56 ± 1.19	200	74.80 ± 1.40	83.61 ± 1.67
IC50	17.54	8.77	IC50	43.10	19.63

Data are presented as mean of three triplicates ± SD. One-way ANOVA was used for data analysis (*n* = 3, *p* < 0.05).

**Table 5:** DPPH radical scavenging activity of C1.

Conc. (μg/mL)	C1	Ascorbic Acid
10	31.55 ± 1.82	62.47 ± 1.02
20	44.65 ± 1.33	85.16 ± 1.99
30	60.03 ± 1.17	93.59 ± 2.10
40	71.93 ± 1.92	96.10 ± 0.87
50	83.61 ± 1.72	99.13 ± 0.45

Data are presented as mean of three triplicates ± SD. One-way ANOVA was used for data analysis (*n* = 3, *p* < 0.05).

**Table 6:** ABTS radical scavenging activity of C1.

Conc. (μg/mL)	C1	Ascorbic Acid
10	40.41 ± 1.53	68.96 ± 1.25
20	54.20 ± 1.29	80.60 ± 1.15
30	65.17 ± 1.61	89.27 ± 1.83
40	78.43 ± 0.89	94.68 ± 1.61
50	85.30 ± 0.93	99.16 ± 0.39

Data are presented as mean of three triplicates ± SD. One-way ANOVA was used for data analysis (*n* = 3, *p* < 0.05).

**Table 7:** Reduction capability activity of C1.

Conc. (μg/mL)	C1	Ascorbic Acid
10	0.193 ± 0.02	0.412 ± 0.01
20	0.281 ± 0.05	0.536 ± 0.04
30	0.379 ± 0.03	0.641 ± 0.06
40	0.502 ± 0.05	0.698 ± 0.03
50	0.618 ± 0.04	0.763 ± 0.02

Data are presented as mean of three triplicates ± SD. One-way ANOVA was used for data analysis (*n* = 3, *p* < 0.05).

**Table 8:** Fe^2+^ ion chelation ability activity of C1.

Conc. (μg/mL)	C1	Ascorbic Acid
10	40.17 ± 1.24	63.58 ± 1.05
20	47.33 ± 1.41	79.69 ± 1.12
30	52.30 ± 1.65	89.37 ± 1.58
40	59.05 ± 1.17	94.48 ± 1.86
50	67.91 ± 1.82	98.08 ± 0.82

Data are presented as mean of three triplicates ± SD. One-way ANOVA was used for data analysis (*n* = 3, *p* < 0.05).

**Table 9:** Lipid peroxidation inhibition capacity activity of C1.

Conc. (μg/mL)	C1	Ascorbic Acid
10	47.89 ± 0.87	55.63 ± 1.20
20	58.04 ± 1.13	63.85 ± 1.54
30	63.71 ± 1.29	79.36 ± 2.01
40	69.63 ± 1.18	88.36 ± 2.15
50	78.10 ± 1.52	95.78 ± 1.08

Data are presented as mean of three triplicates ± SD. One-way ANOVA was used for data analysis (*n* = 3, *p* < 0.05).

**Table 10:** O^2−^ radicals scavenging capacity of Physcion.

Conc. (μg/mL)	C1	Ascorbic Acid
10	59.91 ± 0.99	68.34 ± 1.25
20	70.67 ± 1.38	75.85 ± 1.89
30	78.81 ± 1.62	86.39 ± 1.58
40	83.44 ± 1.26	92.54 ± 1.32
50	89.27 ± 1.51	96.35 ± 1.97

Data are presented as mean of three triplicates ± SD. One-way ANOVA was used for data analysis (*n* = 3, *p* < 0.05).

**Table 11:** NO scavenging capacity of C1.

Conc. (μg/mL)	C1	Ascorbic Acid
10	37.99 ± 1.93	59.99 ± 1.12
20	46.81 ± 1.06	71.26 ± 1.08
30	52.10 ± 1.42	78.63 ± 1.09
40	61.83 ± 1.79	88.14 ± 2.13
50	70.55 ± 1.71	95.78 ± 1.95

Data are presented as mean of three triplicates ± SD. One-way ANOVA was used for data analysis (*n* = 3, *p* < 0.05).

**Table 12:** ADME-related physicochemical parameters of C1.

Predictive Model Parameters		Values
Physicochemical Properties	Molecular Weight	248.26
	Csp3 Fraction	0.12
	Rotatable bonds	1
	H-bond acceptors	5
	H-bond donors	2
	Molar Refractivity	75.25
	Topological polar surface area (TPSA)	83.83 Å²
Lipophilicity	Log Po/w (XLOGP3)	3.04
	Log Po/w (WLOGP)	2.19
	Log Po/w (MLOGP)	0.61
Solubility	Log S (ESOL)	−3.87
	Solubility	3.80 × 10^−2^ mg/mL; 1.34 × 10^−4^ mol/L
	Class	Soluble
Druglikeness	Lipinski (RO5)	Yes; 0 violation
	Ghose	Yes
	Veber	Yes
	Bioavailability Score	0.55
Leadlikness	Rule of three (RO3)	1 alert: quinone_A
	Synthetic accessibility	2.69

Log S = the decimal logarithm of the molar solubility in water, and Log Po/w = the partition coefficient between n-octanol and water. Lipophilicity (Log Po/w) 5, MW 500, H-bond donors 5, and H-bond acceptors 10 are the Lipinski (RO5) criteria. The Log Po/w filter criteria vary from −0.4 to +5.6, the MR from 40 to 130, the MW from 180 to 480, and the number of atoms from 20 to 70. RB 10 and TPSA 1402 are the Veber rule criterion ranges. XLOGP3 3.5, MW 350, H-bond donors 3, H-bond acceptors 3, and RB 3 are among the iRO3 requirements. The synthetic accessibility (SA) score ranges from 1 (very simple) to 10 (extremely difficult) (very difficult).

**Table 13:** Pharmacokinetic parameters of C1.

Pharmacokinetics Parameters	Compounds
GI (HIA) absorption	High
BBB permeant	No
P-GP substrate	No
CYP1A2 inhibitor	Yes
CYP2C19 inhibitor	No
CYP2C9 inhibitor	Yes
CYP2D6 inhibitor	No
CYP3A4 inhibitor	Yes
Log Kp (skin permeation: cm/s)	−4.80 cm/s

**Table 14:** In silico toxicity prediction of C1.

Classification	Target	Prediction
Organ toxicity	Hepatotoxicity	Inactive
Toxicity endpoints	Immunotoxicity	Inactive
Toxicity endpoints	Mutagenicity	Active
Toxicity endpoints	Cytotoxicity	Active
Tox21-nuclear receptor signaling pathways	Aryl hydrocarbon Receptor (AhR)	Active
Tox21-nuclear receptor signaling pathways	Androgen Receptor (AR)	Inactive
Tox21-nuclear receptor signaling pathways	Androgen Receptor Ligand Binding Domain (AR-LBD)	Inactive
Tox21-nuclear receptor signaling pathways	Aromatase	Inactive
Tox21-nuclear receptor signaling pathways	Estrogen Receptor Alpha (ER)	Active
Tox21-nuclear receptor signaling pathways	Estrogen Receptor Ligand Binding Domain (ER-LBD)	Inactive
Tox21-nuclear receptor signaling pathways	Peroxisome Proliferator-Activated Receptor Gamma (PPAR-Gamma)	Inactive
Tox21-Stress response pathways	Nuclear factor (erythroid-derived 2)-like 2/antioxidant responsive element (nrf2/ARE)	Inactive
Tox21-Stress response pathways	Heat shock factor response element (HSE)	Inactive
Tox21-Stress response pathways	Mitochondrial Membrane Potential (MMP)	Active
Tox21-Stress response pathways	Phosphoprotein (Tumor Suppressor) p53	Inactive
Tox21-Stress response pathways	ATPase family AAA domain-containing protein 5 (ATAD5)	Inactive

## Data Availability

The datasets used and/or analyzed during the current study are available from the corresponding author on reasonable request.

## References

[1] Andreote F., Gumiere T., Durrer A. (2014). Exploring Interactions of Plant Microbiomes. Sci. Agric..

[2] Mendes R., Garbeva P., Raaijmakers J.M. (2013). The Rhizosphere Microbiome: Significance of Plant Beneficial, Plant Pathogenic, and Human Pathogenic Microorganisms. FEMS Microbiol. Rev..

[3] Philippot L., Raaijmakers J.M., Lemanceau P., van der Putten W.H. (2013). Going Back to the Roots: The Microbial Ecology of the Rhizosphere. Nat. Rev. Microbiol..

[4] Golinska P., Wypij M., Agarkar G., Rathod D., Dahm H., Rai M. (2015). Endophytic Actinobacteria of Medicinal Plants: Diversity and Bioactivity. Antonie Van Leeuwenhoek.

[5] Kusari S., Verma V.C., Lamshoeft M., Spiteller M. (2012). An Endophytic Fungus from Azadirachta Indica A. Juss. That Produces Azadirachtin. World J. Microbiol. Biotechnol..

[6] Rodriguez R.J., White J.F., Amold A.E., Redman R.S. Fungal Endophytes: Diversity and Functional Roles-Rodriguez-2009-New Phytologist-Wiley Online Library. https://nph.onlinelibrary.wiley.com/doi/10.1111/j.1469-8137.2009.02773.x.

[7] Strobel G., Daisy B. (2003). Bioprospecting for Microbial Endophytes and Their Natural Products. Microbiol. Mol. Biol. Rev..

[8] Jalgaonwala R.E., Mohite B.V., Mahajan R.T. (2011). Natural Products from Plant Associated *Endophytic fungi*. J. Microbiol. Biotechnol. Res..

[9] Berchtold N.C., Cotman C.W. (1998). Evolution in the Conceptualization of Dementia and Alzheimer’s Disease: Greco-Roman Period to the 1960s. Neurobiol. Aging.

[10] De-Paula V.J., Radanovic M., Diniz B.S., Forlenza O.V. (2012). Alzheimer’s Disease. Subcell Biochem..

[11] Collie A., Maruff P. (2000). The Neuropsychology of Preclinical Alzheimer’s Disease and Mild Cognitive Impairment. Neurosci. Biobehav. Rev..

[12] Palmqvist S., Hertze J., Minthon L., Wattmo C., Zetterberg H., Blennow K., Londos E., Hansson O. (2012). Comparison of Brief Cognitive Tests and CSF Biomarkers in Predicting Alzheimer’s Disease in Mild Cognitive Impairment: Six-Year Follow-up Study. PLoS ONE.

[13] Rosenberg P.B., Nowrangi M.A., Lyketsos C.G. (2015). Neuropsychiatric Symptoms in Alzheimer’s Disease: What Might Be Associated Brain Circuits?. Mol. Asp. Med..

[14] Bovio E., Garzoli L., Poli A., Luganini A., Villa P., Musumeci R., Mccormack G.P., Cocuzza C.E., Gribaudo G., Mehiri M. (2019). Marine Fungi from the Sponge Grantia Compressa: Biodiversity, Chemodiversity, and Biotechnological Potential. Mar. Drugs.

[15] Tafinta I.Y., Shehu K., Abdulganiyyu H., Rabe A.M., Usman A. (2013). Isolation and Identification of Fungi Associated with the Spoilage of Sweet Orange (*Citrus sinensis*) Fruits in Sokoto State. Niger. J. Basic Appl. Sci..

[16] Ingkaninan K., Temkitthawon P., Chuenchom K., Yuyaem T., Thongnoi W. (2003). Screening for Acetylcholinesterase Inhibitory Activity in Plants Used in Thai Traditional Rejuvenating and Neurotonic Remedies. J. Ethnopharmacol..

[17] McPhee S., Hodges L.D., Wright P.F.A., Wynne P.M., Kalafatis N., Harney D.W., Macrides T.A. (2007). Anti-Cyclooxygenase Effects of Lipid Extracts from the New Zealand Green-Lipped Mussel, *Perna canaliculus*. Comp. Biochem. Physiol. Part B Biochem. Mol. Biol..

[18] Granica S., Czerwińska M.E., Piwowarski J.P., Ziaja M., Kiss A.K. (2013). Chemical Composition, Antioxidative and Anti-Inflammatory Activity of Extracts Prepared from Aerial Parts of *Oenothera biennis* L. and *Oenothera paradoxa* Hudziok Obtained after Seeds Cultivation. J. Agric. Food Chem..

[19] Amessis-Ouchemoukh N., Madani K., Falé P.L.V., Serralheiro M.L., Araújo M.E.M. (2014). Antioxidant Capacity and Phenolic Contents of Some Mediterranean Medicinal Plants and Their Potential Role in the Inhibition of Cyclooxygenase-1 and Acetylcholinesterase Activities. Ind. Crops Prod..

[20] Miller N.J., Rice-Evans C.A. (1997). The Relative Contributions of Ascorbic Acid and Phenolic Antioxidants to the Total Antioxidant Activity of Orange and Apple Fruit Juices and Blackcurrant Drink. Food Chem..

[21] Gülçin I., Küfrevioglu O.I., Oktay M., Büyükokuroglu M.E. (2004). Antioxidant, Antimicrobial, Antiulcer and Analgesic Activities of Nettle (*Urtica dioica* L.). J. Ethnopharmacol..

[22] Dinis T.C., Maderia V.M., Almeida L.M. (1994). Action of Phenolic Derivatives (Acetaminophen, Salicylate, and 5-Aminosalicylate) as Inhibitors of Membrane Lipid Peroxidation and as Peroxyl Radical Scavengers. Arch. Biochem. Biophys..

[23] Banerjee P., Eckert A.O., Schrey A.K., Preissner R. (2018). ProTox-II: A Webserver for the Prediction of Toxicity of Chemicals. Nucleic Acids Res..

[24] Daina A., Michielin O., Zoete V. (2017). SwissADME: A Free Web Tool to Evaluate Pharmacokinetics, Drug-Likeness and Medicinal Chemistry Friendliness of Small Molecules. Sci. Rep..

[25] Molecular Operating Environment (MOE)|MOEsaic|PSILO. https://www.chemcomp.com/Products.htm.

[26] Manojlovic N., Solujic S., Sukdolak S. (2002). Antimicrobial Activity of an Extract and Anthraquinones from *Caloplaca schaereri*. Lichenologist.

[27] Rutkowska E., Pajak K., Jozwiak K. (2012). Lipophilicity-Methods of Determination and Its Role in Medicinal Chemistry. Acta Pol. Pharm..

[28] Potts R.O., Guy R.H. (1992). Predicting Skin Permeability. Pharm. Res..

[29] Daina A., Zoete V. (2016). A BOILED-Egg to Predict Gastrointestinal Absorption and Brain Penetration of Small Molecules. ChemMedChem.

[30] Roy R.N., Laskar S., Sen S.K. (2006). Dibutyl Phthalate, the Bioactive Compound Produced by Streptomyces Albidoflavus 321.2. Microbiol. Res..

[31] Arnault G., Mony C., Vandenkoornhuyse P. (2022). Plant Microbiota Dysbiosis and the Anna Karenina Principle. Trends Plant Sci..

[32] Rana K.L., Kour D., Sheikh I., Yadav N., Yadav A.N., Kumar V., Singh B.P., Dhaliwal H.S., Saxena A.K. (2019). Biodiversity of Endophytic Fungi from Diverse Niches and Their Biotechnological Applications. Advances in Endophytic Fungal Research: Present Status and Future Challenges.

[33] Marag P.S., Suman A., Gond S. (2018). Prospecting Endophytic Bacterial Colonization and Their Potential Plant Growth Promoting Attributes in Hybrid Maize (*Zea mays* L.). Int. J. Curr. Microbiol. Appl. Sci.

[34] Sukar N.A., Elazab N.T. (2020). Molecular Characterization and Fungicidal Activity of Some Isolated *Endophytic fungi* from Some Wild Plants in Egypt. J. Plant Prot. Pathol..

[35] Gómez O.C., Luiz J.H.H. (2018). Endophytic Fungi Isolated from Medicinal Plants: Future Prospects of Bioactive Natural Products from Tabebuia/Handroanthus Endophytes. Appl. Microbiol. Biotechnol..

[36] dos Reis J.B.A., Lorenzi A.S., do Vale H.M.M. (2022). Methods Used for the Study of Endophytic Fungi: A Review on Methodologies and Challenges, and Associated Tips. Arch. Microbiol..

[37] Sharma D., Pramanik A., Agrawal P.K. (2016). Evaluation of Bioactive Secondary Metabolites from Endophytic Fungus Pestalotiopsis Neglecta BAB-5510 Isolated from Leaves of Cupressus Torulosa D.Don. 3 Biotech.

[38] Pang M.-J., Yang Z., Zhang X.-L., Liu Z.-F., Fan J., Zhang H.-Y. (2016). Physcion, a Naturally Occurring Anthraquinone Derivative, Induces Apoptosis and Autophagy in Human Nasopharyngeal Carcinoma. Acta Pharmacol. Sin..

[39] Lee G., Choi T.W., Kim C., Nam D., Lee S.-G., Jang H.-J., Lee J.-H., Um J.-Y., Jung S.H., Shim B.S. (2012). Anti-Inflammatory Activities of Reynoutria Elliptica through Suppression of Mitogen-Activated Protein Kinases and Nuclear Factor-ΚB Activation Pathways. Immunopharmacol. Immunotoxicol..

[40] Lin Y.-L., Wu C.-F., Huang Y.-T. (2008). Phenols from the Roots of Rheum Palmatum Attenuate Chemotaxis in Rat Hepatic Stellate Cells. Planta Med..

[41] Li F., Xue F., Yu X. (2017). GC-MS, FTIR and Raman Analysis of Antioxidant Components of Red Pigments from Stemphylium Lycopersici. Curr. Microbiol..

[42] Bowen L., Li C., Bin L., Ying T., Shijun L., Junxing D. (2020). Chemical Constituents, Cytotoxic and Antioxidant Activities of Extract from the Rhizomes of Osmunda Japonica Thunb. Nat. Prod. Res..

[43] Comini L.R., Morán Vieyra F.E., Mignone R.A., Páez P.L., Laura Mugas M., Konigheim B.S., Cabrera J.L., Núñez Montoya S.C., Borsarelli C.D. (2017). Parietin: An Efficient Photo-Screening Pigment in Vivo with Good Photosensitizing and Photodynamic Antibacterial Effects in Vitro. Photochem. Photobiol. Sci..

[44] Wen H., Jung H., Li X. (2015). Drug Delivery Approaches in Addressing Clinical Pharmacology-Related Issues: Opportunities and Challenges. AAPS J..

[45] Meng X.-Y., Zhang H.-X., Mezei M., Cui M. (2011). Molecular Docking: A Powerful Approach for Structure-Based Drug Discovery. Curr. Comput. Aided Drug Des..

